# International collaborative project to compare and track the nutritional composition of fast foods

**DOI:** 10.1186/1471-2458-12-559

**Published:** 2012-07-27

**Affiliations:** 1 Global Database Manager, The George Institute for Global Health, PO Box M201, Missenden Rd, Camperdown, NSW, 2050, Australia

**Keywords:** Food composition database, Food industry, Fast food, Monitoring

## Abstract

**Background:**

Chronic diseases are the leading cause of premature death and disability in the world with over-nutrition a primary cause of diet-related ill health. Excess quantities of energy, saturated fat, sugar and salt derived from fast foods contribute importantly to this disease burden. Our objective is to collate and compare nutrient composition data for fast foods as a means of supporting improvements in product formulation.

**Methods/design:**

Surveys of fast foods will be done in each participating country each year. Information on the nutrient composition for each product will be sought either through direct chemical analysis, from fast food companies, in-store materials or from company websites. Foods will be categorized into major groups for the primary analyses which will compare mean levels of saturated fat, sugar, sodium, energy and serving size at baseline and over time. Countries currently involved include Australia, New Zealand, France, UK, USA, India, Spain, China and Canada, with more anticipated to follow.

**Discussion:**

This collaborative approach to the collation and sharing of data will enable low-cost tracking of fast food composition around the world. This project represents a significant step forward in the objective and transparent monitoring of industry and government commitments to improve the quality of fast foods.

## Background

Growing rates of overweight and obesity around the world, in conjunction with a rise in nutrition-related diseases [1,2], has focused attention on the quality of products provided by the food industry [3,4]. In developed countries, and increasingly in developing countries, consumers are purchasing large numbers of meals outside the home, meaning that fast food is contributing substantially to population intakes of energy, fat, sugar and salt [5,6]. Fast food tends to be more energy dense, higher in saturated fat and salt, lower in micronutrients, and eaten in larger portions relative to other foods [7-9]. As a direct consequence, greater consumption of fast food has been associated with an increased risk of both overweight and obesity [10], as well as adverse health outcomes associated with excess body weight. With portion sizes of many fast foods having doubled over the past 50 years [11], it has become even more important to monitor changes in the nutritional content of these products.

In most countries governments have been reluctant to impose additional regulation on food manufacturers to improve the nutritional profile of their products, relying instead on self-regulation or voluntary codes of practice. These approaches have delivered progress in food reformulation in a few countries [12-14] but are weak unless compliance programs are also put in place. Systematic, objective and authoritative monitoring of product formulation in numerous countries could provide additional support to efforts to drive change in the composition of fast foods. Accordingly, The Food Monitoring Group was established in 2010 with the aim of objectively tracking changes to the nutritional composition of the food supply. An initial protocol for monitoring packaged food products has been developed [15] and a series of reports have emanated [16-18]. This paper now describes a protocol for comparing and monitoring the nutritional composition of fast food products around the world.

### Overall goal and objectives

The overall goal of this project is to collate nutrient composition data for fast foods in different countries with the objective of supporting efforts to improve the quality of products provided by the global fast food industry. This will be done by collecting each year, information about product composition in a standardized format for major fast food chains in a large number of different countries. The primary outcome measures to be assessed will be energy content, saturated fat, sugar, sodium, and serving size, in line with the World Health Organization’s Global Strategy on Diet, Physical Activity and Health [19]. There will be three main objectives:

1. To compare the mean levels and ranges of the primary outcome measures in each fast food category between countries

2. To compare the mean levels and ranges of primary outcome measures for fast food categories between companies

3. To track changes over time in the mean levels and ranges of the primary outcome measures in fast food categories by country and by company.

## Methods/design

This project will comprise annual surveys of fast food products in countries around the world with the goal of documenting the composition of the main products available for purchase in each major fast food chain in each country. The same basic methodology will be applied in each country to enable robust comparisons at baseline and reliable tracking of changes in product composition over time.

### Countries to be included

The goal is to include a broad geographic coverage of countries. There will be no restriction on the number of countries that can participate in this project although in practice the availability of data and resources will initially limit the countries involved.

### Companies and products to be included

A fast food chain will be defined as an outlet that sells food products that are ready-to-eat, sold in servings with standard content and size, and not in their final package before arriving at the outlet. In the first instance we will seek to include the ten fast food chains with the most outlets in each country. If resources permit we will further seek to include all fast food chains with 20 or more outlets in the participating country.

In reporting of project findings the description of sampling methods in each country will be outlined, the likely completeness of coverage achieved described and the potential for bias in the data collection process detailed.

### Data sources

Depending upon the resource available, collaborating countries will determine the most feasible data source. These may include:

· *Fast food companies* - if possible countries will be encouraged to obtain nutritional information directly from fast food companies to ensure the data are as up to date as possible

· *Websites* – most trans-national fast food companies provide nutritional information for their products on the company website

· *Direct chemical analysis* – in countries where nutrient information is not available for fast food products, and where resource is available, data may be obtained through direct chemical analysis of the products. It is likely that in this situation resources will limit the number of fast food chains and products that can be targeted. If analyzed data are used this may also be used in quality control of data from other sources

· *Pamphlets/tray liners/other packaging information collected in*-*store* – in some countries information on nutrient content may be available on materials available in-store such as tray liners and pamphlets, as well as the product packaging.

The information included in the database will be in English.

### Nutrient data to be collected

Collaborating countries will be encouraged to adopt one of the following strategies for data collection every year, depending upon the level of resource available:

· *Nutrient information for all products from all selected fast food chains*– If adequate resources are available this is the preferred option. Major fast food outlets in each country will be identified, a full listing of all foods for sale recorded and the primary variables sought for each product. Where nutrient information is not available, information on the company name and product name will still be recorded.

· *Focus on data for selected fast food chains* – Where resources are limited initial efforts may be restricted to specific fast food chains. For example, information may be more readily available from large multinational fast food chains than local chains. Collaborators will be encouraged to collect information from as many major chains as possible, in line with the company inclusion criteria.

The variables that will be sought for each fast food item are indicated in Table 1. If full data are not available values will be recorded as missing. In particular, products for which only company name and product name are available with no data on nutritional content will be recorded to highlight the absence of data. Wherever resources allow, the data entry process will be checked by selecting a random sample of 5 % of entries from each country and having a second researcher compare the information in the database against the original source. Data will be entered into a password-protected online database with the data source recorded for each entry. Data entry will be done either manually product by product, or by upload of data from another electronic source. Central management will be provided by The George Institute in Sydney, Australia with data collection materials made available to collaborators as required.

**Table 1:** Variables to be collected‡ and format

**Primary**	**Format**
Country	country where data are collected
Fast food category	refer to Table 2
Fast food sub-category (major)	refer to Table 2
Company name	as per data source
Product name	as per data source
Serving size	grams or millilitres
Energy	kilojoules or kilocalories / 100 grams or 100 millilitres
Saturated fat	grams / 100 grams or 100 millilitres
Total sugars	grams / 100 grams or 100 millilitres
Sodium*	milligrams / 100 grams or 100 millilitres
Data source	NIP, MANUF, WEB, DATAB, OTHER
Date of data collection	date (dd/mm/yyyy)
**Secondary**^**¶**^	
Total fat	grams / 100 grams or 100 millilitres
Trans fat	grams / 100 grams or 100 millilitres
Monounsaturated fat	grams / 100 grams or 100 millilitres
Polyunsaturated fat	grams / 100 grams or 100 millilitres
Protein	grams / 100 grams or 100 millilitres
Carbohydrate	grams / 100 grams or 100 millilitres
Dietary fibre	grams / 100 grams or 100 millilitres
Sub-category (minor)	as defined for each country
Ingredients list	Listing of ingredients
Price	cost of product per item
Promoted as healthy option	Yes/No – promoted by food manufacturer as small portion, lite option, or healthier option
Notes	as deemed important by each collaborating country

NIP, nutrition information panel on product packaging; MANUF, direct from manufacturer; WEB, direct from internet site; DATAB, from external branded database.

*it will also be possible to submit data as salt in grams / 100 grams or 100 millilitres.

‡ countries will be required to indicate if the definition for a nutrient varies from that in the protocol.

^¶^ additional variables can be collected by each country as required (e.g. calcium).

### Categorization of fast foods

The definitions used for these product categories are based on those utilized for prior reports [16,20] which were in turn derived from the categorizations commonly used by the fast food industry (Table 2). The overarching goal for the categorization system is that it be broadly applicable internationally and reflect both industry practices and consumer purchasing patterns. This will enable reporting that is easily interpretable by industry, government, consumers and other stakeholders. Some additional food categories may be identified as further countries participate and there will be scope to add to the initially defined categorization system if required. While this may increase complexity it will enable appropriate flexibility in the collection of data and the reporting of results.

**Table 2:** Fast food categorization system

**Fast food category**	**Food sub-category (major)**	**Food sub-category (minor)**	**Description**
Asian	Thai		Thai take-away meals and products
	Chinese		Chinese take-away meals and products
	Indian		Indian take-away meals and products
	Sushi and rice-paper rolls		All sushi and rice-paper rolls
	Other		Other Asian take-away products and meals not in above categories
Beverages	Juice		Fruit and vegetable juices, pure fruit smoothies
	Milkshakes/ smoothies		Milkshakes, thickshakes, milk-based smoothies and other milk-based drinks
	Water		Plain and flavoured waters
	Soft drink	Sugar-sweetened	Sugar-sweetened soft drinks
		Sugar free	Artificially-sweetened soft drinks
	Tea/coffee/hot chocolate		Tea, coffee, hot chocolate, and iced variations
	Other		Other take-away beverages
Breakfast	Savoury		Breakfast rolls/wraps/sandwiches, hot breakfasts, hash brown
	Sweet		Pancakes/hotcakes
	Other		Plain English muffins, bagels, yoghurt, cereal
Burgers	Beef burgers		All beef-based burger products (excluding sandwiches)
	Fish burgers		All fish-based burger products (excluding sandwiches)
	Chicken burgers		All chicken-based burger products (excluding sandwiches)
	Vegetarian burgers		All vegetarian burger products (excluding sandwiches)
Chicken			Fried/grilled/roasted chicken, nuggets, hot wings
Dessert			Dessert products
Dressings/ condiments	Sweet		Dessert sauces, sweet spreads
	Savoury		Salad dressing, croutons, savoury sauces and spreads, gravy
Other			Ribs, boxed meals etc.
Pasta			Pasta dishes
Pizza	Meat-based toppings		Take-away pizza products with meat-based toppings
	Seafood toppings		Take-away pizza products with seafood-based toppings
	Vegetarian toppings		Take-away pizza products with vegetable-based toppings
Salads	Garden/plain salads		Garden salads or other plain salads
	Salads with meat		Salad products with beef, chicken, lamb or fish
Sandwiches	Beef-based sandwiches		Beef-based sandwiches, wraps etc. (excludes burger-bun products)
	Chicken-based sandwiches		Chicken-based sandwiches, wraps etc. (excludes burger-bun products)
	Fish-based sandwiches		Fish-based sandwiches, wraps etc. (excludes burger-bun products)
	Vegetarian sandwiches		Vegetarian sandwiches, wraps etc. (excludes burger-bun products)
	Other sandwich products		All other sandwich products
Seafood			All seafood products
Sides	Fries		Salted and unsalted fries
	Other		All sides (excluding fries) such as onion rings, vegetables, garlic bread etc.
Soup			Soup products
Cakes, muffins and pastry	Cakes		Sweet cake products including sweet muffins
	Muffins		Savoury muffins
	Sweet pastry-based products		Pastry-based products such as danishes, tarts etc.
	Savoury pastry-based products		Savoury-based products such as empanadas, pies etc.
Other cereal-based products	Tacos		Tacos and other wheat/maize-based products
	Kebabs		Kebab products

### Analysis and reporting of data

Analyses will initially focus on the primary outcome measures (Table 1). There will be tabulations that summarize the number of products in each category and the completeness of the data overall, by country and by company. Mean levels (and ranges) for all nutrient values will be determined for the same groupings. Primary analyses will be reported per 100 g with additional estimates made per serve. Where values are only reported per serve, efforts will be made to also obtain values per 100 g or else calculated on the basis of the serving size. Mean values of nutrients will be compared between companies, between countries and over time.

### Current status

Data for six countries (Australia, France, UK, USA, New Zealand and Canada) have been entered into the central database for proof of concept comprising full nutritional information for >2,000 fast food products in 2011 [21]. We anticipate the addition of data from as many countries again in the next 12 months and further increases each year thereafter. The challenge is likely to be in obtaining data describing the nutrient content of fast foods manufactured by smaller companies and companies operating in developing countries where nutrition information is not readily available. In these circumstances the efforts of local collaborators will be essential and direct chemical analysis may be required to determine composition.

### Management, data sharing and authorship

The project will be managed on a day-to-day basis by an operational Secretariat based at The George Institute for Global Health in Sydney. High-level decisions about the direction of the initiative will be made by the Management Committee which will be comprised of one nominated senior representative from each participating country (members listed in Acknowledgements). The Management Committee member for each country may also nominate other individuals involved in the database as members of The Food Monitoring Group.

Each contributing country will have access to summary data from all countries as well as full access to their own data. Collaborators will be free to independently analyze and publish communications based upon the data they have contributed. Analyses and outputs involving data from two or more participating countries will require the agreement of each Management Committee member who will be responsible for sign off on each use of the data from their country. There will be a number of primary publications involving all the countries in the collaboration and the Secretariat will take responsibility for ensuring that agreement is obtained from all parties for these. Authorship of these primary publications will be in the name of the collaborative group (The Food Monitoring Group). Authorship of publications involving a limited number of countries will be at the discretion of the Management Committee members involved.

Management Committee members will be free to distribute their own dataset to other collaborators in this initiative and groups outside the collaboration. The Secretariat will not provide datasets from any country to a third party and collaborators from one country will not have the capacity to pass on the dataset of another country. External access to the full datasets will only be provided if all Management Committee members agree. In general the principle underlying the distribution of information from the project will be that it be shared freely amongst groups with public health goals with restrictions on sharing limited primarily to ensure quality of analysis and outputs. This will include industry groups who may be provided with reports through collaborations established with the Management Committee members as part of their efforts to improve the quality of the food supply.

## Discussion

This initiative represents the first coordinated effort to objectively and systematically quantify the characteristics of high volume fast foods sold around the world. It is anticipated that the ready availability of such data will support global fast food companies and governments in their efforts to improve the quality of the food supply [22]. In particular we hope the project can be used to drive improvements in the average composition of fast foods around the world, which even if small, have great potential to deliver significant health gains because so many people eat fast food so often [23].

Early outputs from the project will allow primarily for between country and between company comparisons of fast foods currently on the market. These analyses will set the baseline against which future progress in improving the nutritional quality of fast foods can be recorded. Pilot work using fast food nutrient data from six countries and six large multinational fast food outlets has shown the large variation in fast food composition between product categories, countries and companies [21] and a more detailed analysis of the Australian data showed that there were 5-, 10- and even 20-fold differences in salt content between comparable products [16]. This level of variation is unlikely to be required for technical reasons and suggests significant potential for reformulation towards healthier compositions. The international comparisons also showed that apparently identical products sold by the same company in different markets can have markedly different compositions between countries.

A primary objective of the project is to collect data in the same format across multiple jurisdictions over time. The use of a standard protocol will make direct comparisons between diverse regions of the world possible and allow for robust monitoring of changes in the composition of products. There is significant need for an independent third party to take on this role because the fast food sector makes many commitments to improving its products but there are few countries in which governments or their agencies monitor the impact of these pledges.

The project currently involves a limited number of countries and is not globally representative in its initial membership. It is anticipated that additional countries will become involved as the project progresses and the protocol has been designed to enable this. The absence of available nutrition information for fast food products in some countries may limit the analyses that can be done. However, highlighting the absence of data for particular countries and particular companies will be an important secondary output from this project and will be used to drive policy changes towards greater transparency. The absence of sales data that define the numbers of each product sold will also be a significant limitation since a number of companies have introduced healthier menu items but have not made available data about the volume of the product sold [24].

There is also the possibility that perceived inaccuracies in the food composition data reported by the companies will undermine the integrity of the project. It will be impossible to directly analyze the levels of all nutrients in all products in the database, although there are a number of countries in which a sample of foods will be tested in this way. As such it will be possible to make some quantification of the extent to which systematic or random errors might influence the project conclusions. In conjunction with the observation that many of the larger chains already base their nutritional reporting on direct analysis done by credible parties external to their organizations it is not anticipated that the quality of the data will be a major issue.

In conclusion, this project will provide new information about the composition of fast foods around the world which will be used to support efforts to achieve progressive, manageable, across-the-board reformulation of fast food products around the globe. Sustained small-to-moderate improvements in the food supply would reap significant public health gains and avert much premature chronic disease. With the rates of non-communicable diseases such as obesity, type 2 diabetes and cardiovascular disease increasing worldwide, there is a strong argument for renewed efforts to achieve population-wide reductions in sodium, saturated fat, sugar and energy consumption.

## Competing interests

Food Monitoring Group Management Committee: Elizabeth Dunford is the research officer and Bruce Neal is the Chair of the Australian Division of World Action on Salt and Health. Graham Macgregor is the Chairman of World Action on Salt and Health. Elizabeth Dunford has received funding from the World Health Organisation (WHO) to develop a tool for monitoring sodium content in foods; Sebastien Czernichow is a board member and consultant for Sanofi; he is a consultant for Unilever and received payment for developing education presentations; he has received payment for lectures from Servier. Cliona Ni Mhurchu is a consultant for Food Standards New Zealand; she is a member of the Food and Nutrition Working Group for the Heart Foundation of New Zealand; she has been reimbursed for travel expenses by the International Life Sciences Institute. Bruce Neal is a consultant and member of the boards for Roche Diagnostics, Takeda and PepsiCo; he has received payment for lectures or reimbursement for travel from Amgen, AstraZeneca, Glaxo- SmithKline, Novartis, PepsiCo, Pfizer, the Pharmacy Guild of Australia, Sanofi Aventis, Servier and Tanabe; his institution has received funding from the Australian Food and Grocery Council, Bupa Australia, Johnson and Johnson, Merck Schering Plough, Roche Diagnostics, servier and United Healthcare Group. No other competing interests were declared by members of the Food Monitoring Group.

## Author’s contributions

ED wrote the first draft of the manuscript with input and review from all other authors (members of The Food Monitoring Group). All authors read and approved the final manuscript.

## Sources of support

E Dunford is supported by a Sydney Medical School Foundation scholarship and B Neal by an Australian Research Council Future Fellowship.

## Pre-publication history

The pre-publication history for this paper can be accessed here:

http://www.biomedcentral.com/1471-2458/12/559/prepub
